# Gut microbiota imbalance in colorectal cancer patients, the risk factor of COVID-19 mortality

**DOI:** 10.1186/s13099-021-00466-w

**Published:** 2021-12-04

**Authors:** Changjing Cai, Xiangyang Zhang, Yihan Liu, Edward Shen, Ziyang Feng, Cao Guo, Ying Han, Yanhong Ouyang, Hong Shen

**Affiliations:** 1 Department of Oncology, Xiangya Hospital, Central South University, Changsha, Hunan China; 2 Key Laboratory for Molecular Radiation Oncology of Hunan Province, Xiangya Hospital, Central South University, Changsha, China; 3 Department of Life Science, McMaster University, Hamilton, ON L8S 4L8 Canada; 4 Department of Pathology, University of Pittsburgh School of Medicine, Pittsburgh, PA USA; 5 Department of Emergency, Hainan General Hospital, Hainan Affiliated Hospital of Hainan Medical University, 19 Xiuhua Road, Haikou, 570311 Hainan China; 6 National Clinical Research Center for Geriatric Disorders, Xiangya Hospital, Central South University, Changsha, China

**Keywords:** Gut microbiota, Colorectal cancer, COVID-19, Mortality, Prognosis

## Abstract

**Background:**

COVID-19 pandemic is sweeping across the world. Previous studies have shown that gut microbiota is associated with COVID-19, and operational taxonomic unit (OTU) composed of Blautia genus, Lactobacillus genus, and Ruminococcus genus of Firmicutes is correlated with the severity of COVID-19. Gut microbiota imbalance in colorectal cancer patients may lead to the variation of OTU.

**Results:**

Based on the GMrepo database, the gut microbiota of 1374 patients with colorectal neoplasms and 27,329 healthy people was analyzed to investigate the differences in the abundance of microbes between colorectal neoplasms patients and healthy people. Furthermore, We collected feces samples from 12 patients with colorectal cancer and 8 healthy people in Xiangya hospital for metabolomic analysis to investigate the potential mechanisms. Our study showed that the abundance of Blautia and Ruminococcus was significantly increased in colorectal neoplasms, which may increase the severity of COVID-19. The gender and age of patients may affect the severity of COVID-19 by shaping the gut microbiota, but the BMI of patients does not.

**Conclusions:**

Our work draws an initial point that gut microbiota imbalance is a risk factor of COVID-19 mortality and gut microbiota may provide a new therapeutic avenue for colorectal cancer patients.

**Supplementary Information:**

The online version contains supplementary material available at 10.1186/s13099-021-00466-w.

## Introduction

Since being firstly reported in December 2019, COVID-19 has been rapidly spreading around the world and bringing about unfolding health and socioeconomic impacts. On February 26, 2021, the number of patients infected with COVID-19 worldwide has reached 112,981,257 and the number of deaths has reached 2,507,271 [1]. Accumulating evidence indicated that gut microbiota is related to COVID-19 infection [2–4]. ACE2 is the receptor of COVID-19, and also plays an important role in curbing intestinal inflammation [4, 5]. ACE2 is expressed in both respiratory and digestive tracts, and gut microbiota is closely related to the expression level of ACE2 [6, 7]. Besides, metabolites of gut microbiota are also important in the regulation of the human immune system, which may be related to the cytokine storm produced by COVID-19 [8]. Previous studies of COVID-19 have found that a large proportion of patients developed intestinal symptoms, which further suggests that gut microbiota may be related to COVID-19 infection [9].

Previous studies showed that the condition of COVID-19 patients during hospitalization was closely related to the abundance of gut microbiota, and firmicutes might play an important role. Gou et al. found [10] that the Blautia genus, Lactobacillus genus, and Ruminococcus genus of Firmicutes in the gut microbiota of COVID-19 patients is correlated with the expression of multiple inflammatory molecules. Meanwhile, the operational taxonomic unit (OTU) composed of these three genera is correlated with the severity of COVID-19.

COVID-19 patients with cancer, including colorectal cancer (CRC), have a higher risk of severe events than their counterparts who do not have cancer, which may be associated with advancing age, male sex, but its relationship to gut microbiota need to be further researched [11, 12]. It is more difficult to treat COVID-19 patients with cancer than others because of their complex immune environment [13, 14], and gut microbiota may provide a new therapeutic avenue for these patients. Previous studies showed that the occurrence, development, and treatment of a variety of cancers, including colorectal cancer, are strongly related to gut microbiota [15–18]. The gut microbiota spectrum of colorectal cancer patients is significantly different from that of the healthy control group, with the increase of Fusobacterium nucleatum, Parvimonas, Peptostreptococcus, Porphyromonas, and Prevotella genera [19–26]. Also, several studies indicated that the abundance of Blautia [27, 28] and Ruminococcus [29, 30] changed in the gut microbiota of colorectal cancer patients, and changes in the abundance of these Microbes may affect the severity of COVID-19 disease. However, the correlation between the gut microbiota of tumor patients and COVID-19 remains to be studied.

In this study, the public database (GMrepo) was used to analyze the differences in the abundance of microbes in 1374 patients with colorectal neoplasms and 27,329 health control people. Meanwhile, the data of Blautia Genus, Lactobacillus Genus, and Ruminococcus Genus were screened out for subgroup analysis of age, sex, and BMI. Feces samples from 12 patients with colorectal cancer and 8 healthy people were collected for metabolomic analysis to study the relationship between microbe abundance and inflammatory factors in patients with colorectal cancer. This study suggests that changes in the microbiota of patients with colorectal cancer may exacerbate the condition of patients with COVID-19, and that gut microbiota may be a potential therapeutic target.

## Methods

### Dataset source and preprocessing

#### GMrepo database

GMrepo (data repository for Gut Microbiota) is a database of curated and consistently annotated human gut metagenomes. The clinical features, abundances, and prevalence were retrieved from GMrepo. All 1374 Colorectal Neoplasms patients and 27,329 Health people were included (Additional file 1: Table S1).

#### Gene-Expression Omnibus (GEO) database

A public expression dataset with gut microbiota and cytokines about colorectal cancer patients was gathered from GEO. GSE136682 [18] was selected and downloaded using GEOquery.

### Validation Cohort and fecal sample collection

#### Sequence Read Archive (SRA) database

To further validate our results, gut microbiota about CRC patients and healthy control was downloaded from SRA with accession numbers SRP005150 [31] and SRP149108 [32]. SRP005150 contains 102 samples, of which 46 samples are CRC patients. SRP149108 contains 23 samples, of which 11 samples are CRC patients. Student’s t-test was used to analyze the two datasets.

Furthermore, a prospective cohort was performed. Fecal samples from 12 colorectal cancer patients and 8 Health people were collected in Xiangya Hospital of Central South University from June 2020 to July 2020. The proportion of male patients and elderly patients (> 65) in both groups was 50%. The clinical data including age, gender, and pathological reports were obtained from the electronic medical records (EMR) system (Additional file 1: Table S2). This study was reviewed and approved by the Xiangya Hospital Medical Ethics Committee of Central South University. All qualified stool samples were self-sampled and immediately sent to the laboratory, placed in frozen pipes, and stored at -40℃ for further testing.

### Metabolomics analysis

A liquid chromatography–mass spectrometry (LC–MS) system (Q Exactive Orbitrap, Thermo Fisher Scientific, USA) was utilized to analyze the metabolomics of the fecal samples of Validation Cohort in positive and negative ion modes. 50 mg fecal sample was placed into an Eppendorf (EP) tube and added with extraction solvent (acetonitrile methanol–water, 2:2:1) 1000μL, then vortexed for the 30 s, homogenized shook for 4 min at 45 Hz, and repeated 3 times. Then the samples were stored at -20℃ for 1 h, and centrifugation at 12,000 RPM, 4℃ for 15 min. The obtained supernatant was transferred to LC–MS bottles and stored at 80℃, followed by UHPLC-QE Orbitrap/MS analysis. Mixing the supernatant of all samples evenly as quality control (QC) sample. An ultra-high-performance liquid chromatography (UHPLC) system was subsequently used for LC–MS/MS analysis.

### Statistics analysis

The final Metabolomics dataset containing the information of peak number, sample name, and normalized peak area was imported to the SIMCA16.0.2 software package (Sartorius Stedim Data Analytics AB, Umea, Sweden) for multivariate analysis. To visualize group separation and find significantly changed metabolites, supervised orthogonal projections to latent structures-discriminate analysis (OPLS-DA) were applied. Then, sevenfold cross-validation was performed to calculate the value of R2 and Q2, and 200 times permutations were further conducted. We calculated the correlation coefficient of the quantitative value of importantly changed metabolites using the Pearson method and presented it in the form of a thermal map. Besides, to conduct pathway abundance analysis, KEGG databases (http://www.genome.jp/kegg/) and MetaboAnalyst (http://www.metaboanalyst.ca/) were used. The student’s t-test was used to calculate the P-value. R (version 3.6.3) and ggplot and GEO query were used for data analysis. The metabolites with VIP (variable importance in the projection) > 1 and p < 0.05 were considered as importantly changed metabolites.

## Results

### The abundances of Blautia genus and Ruminococcus genus in colorectal neoplasms patients are higher than health control

**Fig. 1:**
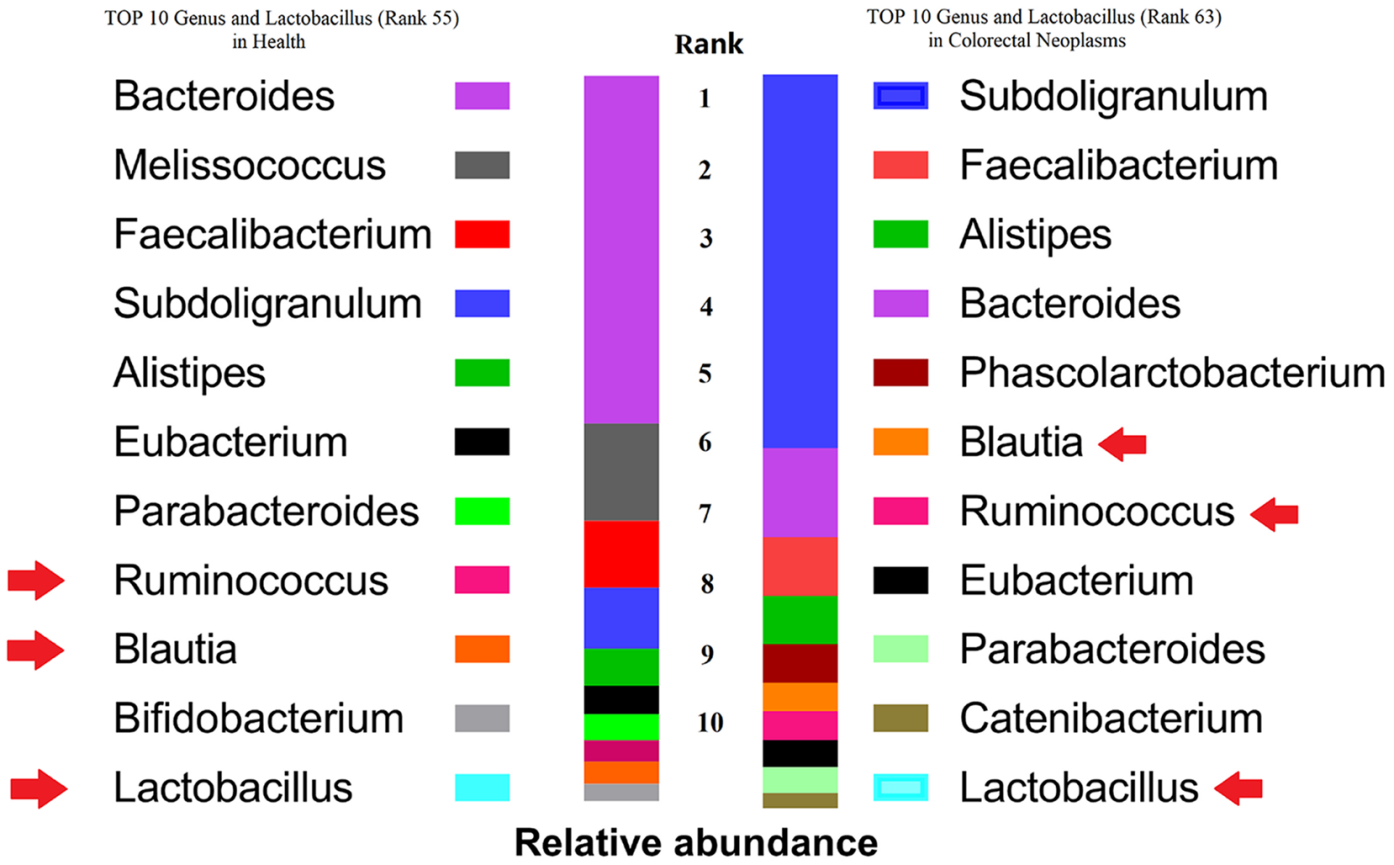
The abundance of Gut microbiota in colorectal cancer patients and healthy control controls based on Gmrepo Database. Blautia and Ruminococcus rank significantly higher in colorectal neoplasm patients than in healthy control people

To investigate the differences between colorectal neoplasms patients' gut microbiota and healthy controls, data from the Gmrepo database was used. In the colorectal cancer group, the 10 bacterial genera with the highest abundance are Subdoligranulum, Faecalibacterium, Alistipes, Bacteroides, Phascolarctobacterium, Blautia, Ruminococcus, Eubacterium, Parabacteroides, Catenibacterium. In the health group, the top 10 are Melissococcus, Faecalibacterium, Subdoligranulum, Bacteroides, Alistipes, Eubacterium, Parabacteroides, Ruminococcus, Blautia, Bifidobacterium. Lactobacillus genus ranks 63 in colorectal neoplasms patients and 55 in healthy people. Blautia and Ruminococcus rank significantly higher in colorectal neoplasm patients than in health control people (Fig. 1).

**Fig. 2:**
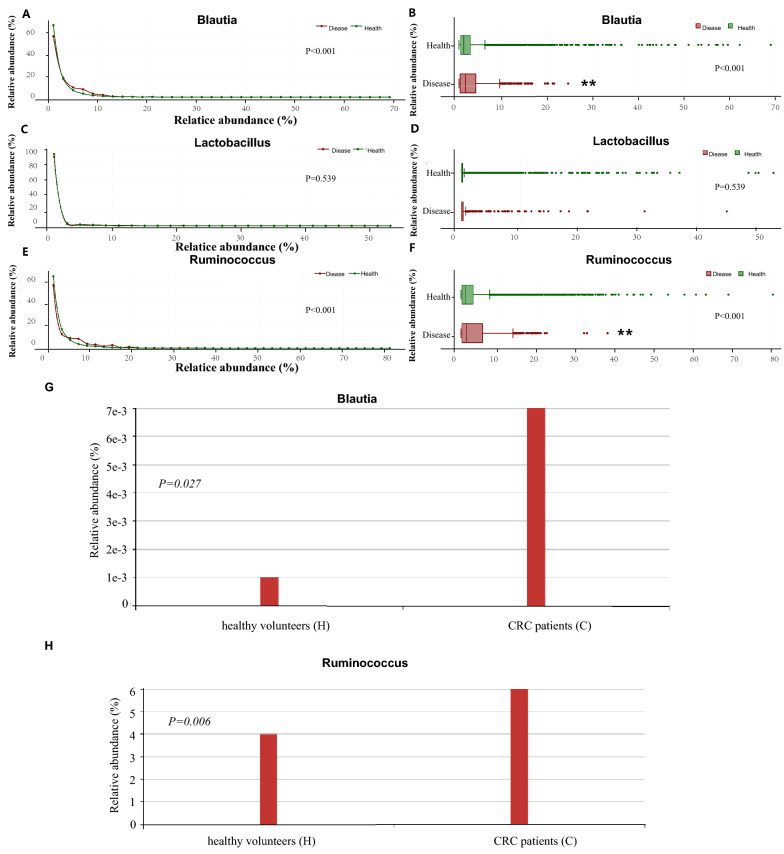
**A** The linear graph showed the abundance of Blaautia in colorectal neoplasm patients and healthy people. **B** The box plot showed the abundance of Blaautia in colorectal neoplasm patients and healthy people, which was significantly increased in colorectal neoplasm patients. **C** The linear graph showed the abundance of Lactobacillus in colorectal neoplasm patients and healthy people. **D** The box plot showed the abundance of Lactobacillus in colorectal neoplasm patients and healthy people. **E** The linear graph showed the abundance of Ruminococcus in colorectal neoplasm patients and healthy people. **F** The box plot showed the abundance of Ruminococcus in colorectal neoplasm patients and healthy people.** p < 0.01. **G** The relative abundance of Blautia of CRC patients and control in SRP005150. **H** The relative abundance of Ruminococcus of CRC patients and control in SRP149108

The differences in abundance levels of Blaautia, Ruminococcus, and Lactobacillus were further analyzed and compared. Abundance data of 1374 colorectal neoplasms patients and 27,329 health people were analyzed (Additional file 1: Table S1), and results showed that median values of the abundance of colorectal neoplasms vs. health were respectively: Blautia (1.47 vs. 1.11, P < 0.001), Ruminococcus (1.44 vs. 1.13, P < 0.001), Lactobacillus (0.0347 vs. 0.0314, P = 0.539). Our study showed that the abundance of Blaautia and Ruminococcus in colorectal neoplasm patients was significantly increased (Fig. 2A–D), but there was no significant difference in the abundance of Lactobacillus (Fig. 2E, F).

To validate the results, two other datasets were analyzed. Analysis of data in SRP005150 showed that Blaautia enrichment was different between colorectal cancer patients and healthy controls (P = 0.027) (Fig. 2G). Data analysis in SRP149108 showed that the enrichment degree of Ruminococcus was different between colorectal cancer patients and healthy controls (P = 0.006) (Fig. 2H).

### The correlation of gut microbiota and clinical features in colorectal neoplasms

**Fig. 3:**
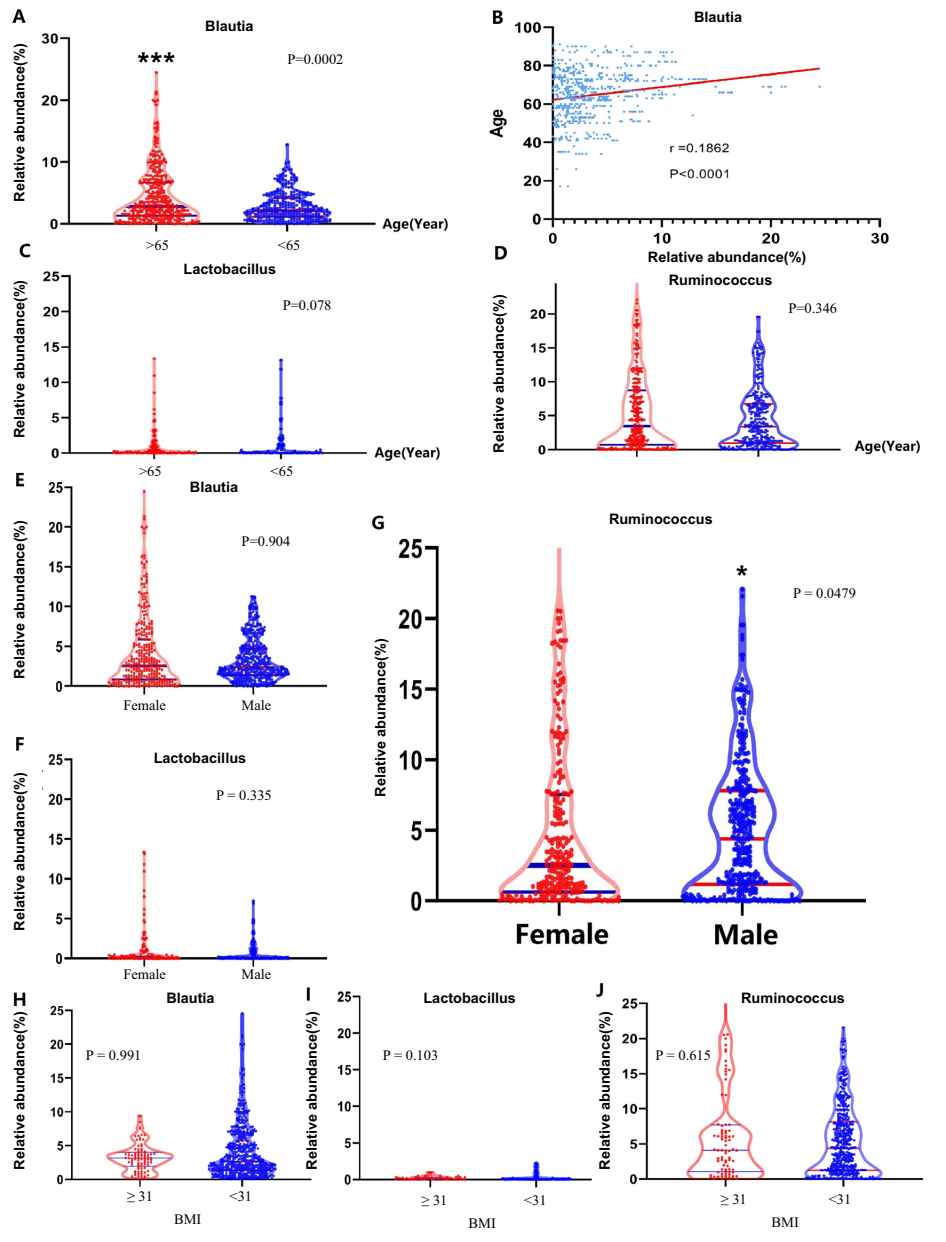
The relative abundance of Blautia, Lactobacillus, and Ruminococcus of colorectal neoplasm patients is divided by age, gender, and BMI. **A** The abundance of Blautia in patients divided by age was shown, and Blautia Genus has a higher abundance in older patients (p < 0.0001). **B** The abundance of Blautia was positively correlated with the age of patients (r = 0.1862, p < 0.0001). **C** The abundance of Lactobacillus in patients divided by age was shown. **D** The abundance of Ruminococcus in patients divided by age was shown. **E** The abundance of Blautia in patients divided by gender was shown. **F** The abundance of Lactobacillus in patients divided by gender was shown. **G** The abundance of Ruminococcus in patients divided by gender was shown, and Ruminococcus has a higher abundance in males (p < 0.05). **H** The abundance of Blautia in patients divided by BMI was shown. **I** The abundance of Lactobacillus in patients divided by BMI was shown. **J** The abundance of Ruminococcus in patients divided by BMI was shown. * p < 0.05, *** p < 0.001

To investigate the relationship between clinical characteristics and Blautia Genus, Lactobacillus Genus, and Ruminococcus Genus, the data of gender, age, and BMI were selected for subgroup analysis (Additional file 1: Table S2). In terms of age, previous studies showed that 65 years of age is a risk factor for COVID-19 mortality [33]. We used 65 as the cut-off point. Abundance of gut microbiota of > 65 vs. < 65 are respectively: Blautia(2 .814 vs. 2.168, P = 0.0002, Fig. 3A), Lactobacillus (0.155 vs. 0.059, P = 0.078, Fig. 3C), Ruminococcus (3.467 vs. 3.377, P = 0.346, Fig. 3D). In addition, the correlation between Blaautia and age was further analyzed, and the results showed that they were positively correlated (Cor. = 0.186, P < 0.0001, Fig. 3B). These results show that in older patients, Blautia Genus has a higher abundance.

Previous studies found that gender is also a risk factor for COVID-19 [34, 35]. Abundance of gut microbiota of Female vs. Male are respectively: Blautia(2.579 vs. 2.404, P = 0.904, Fig. 3E), Lactobacillus(0.164 vs. 0.080, P = 0.335, Fig. 3F), Ruminococcus (2.536 vs. 4.407, P = 0.0479, Fig. 3G). These results indicate that male patients have a higher abundance of Ruminococcus genus.

**Fig. 4:**
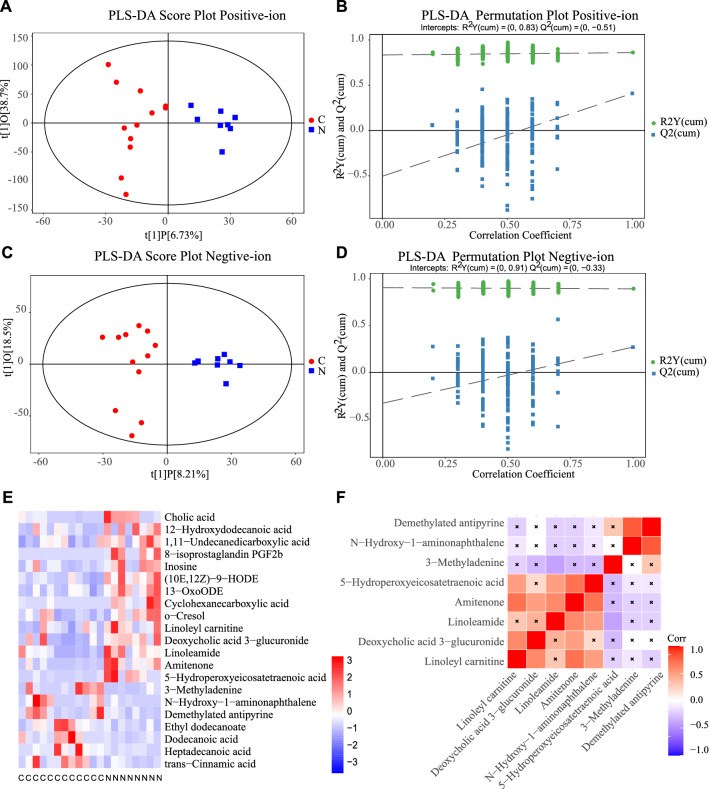
**A** PLS-DA score plot of the control and cancer patients of positive ions was shown based on LC–MS technology. **B **The permutation plot of positive ions (200 times, R2 = 0.83, Q2 =  − 0.51) was conducted and shown. **C** PLS-DA score plot of the control and cancer patients of negative ions was shown based on LC–MS technology. **D** The permutation plot of positive ions (200 times, R2 = 0.83, Q2 =  − 0.51) was conducted and shown. **E** The heat map exhibited the differently expressed substances detected in colorectal cancer patients. **F** The thermodynamic chart showed the expressions of several substances are correlated. Red blocks represent positive correlation, blue blocks represent the negative correlation. And the deeper the color, the stronger the correlation. Non-significant correlations were marked with crosses inside of the boxes

Previous studies suggested that BMI was also associated with the fatality of COVID-19, and we divided patients into two groups using BMI = 31 as a cutoff point [36]. Abundance of gut microbiota of BMI > 31 vs. BMI < 31 are respectively: Blautia (3.162 vs. 2.635, P = 0.991, Fig. 3H), Lactobacillus (0.165 vs. 0.0474, P = 0.103, Fig. 3I), Ruminococcus (4.122 vs. 4.367, P = 0.615, Fig. 4J). Therefore, this study found no significant differences in Blautia genus, Lactobacillus genus, and Ruminococcus genus in patients with different BMI.

### Expression of pro-inflammatory cytokines in intestinal samples from mice gavaged by feces from colorectal cancer patients or healthy controls

To investigate the correlation between cytokines and gut microbiota, we found a relevant dataset in the GEO database (GSE136682) and collected most of the cytokines which were reported related to COVID-19 [37–39]. Based on GSE136682 data, we found that IL-1β and IL-6 were increased in the intestinal tumor of mice after gavage administration by feces from colorectal cancer patients compared to their counterparts of mice fed by feces from healthy people (Additional file 1: Figure S1). Meanwhile, previous studies showed that patients with COVID-19 were in a pro-inflammatory status with high levels of IL-1β and other cytokines [40], and high levels of IL-6 and TNF-α were observed in COVID-19 patients requiring intensive-care-unit hospitalization [41]. Hence, we speculate that increased IL-1β and IL-6 may worsen the disease in colorectal cancer patients with COVID-19.

### Metabolomics differences between colorectal cancer patients and healthy control group

Metabolomic data used in this study were obtained from 12 patients with colorectal cancer and 8 health control people in Xiangya Hospital. In addition to their health status, they were classified by sex and age. PLS-DA analysis (Fig. 4A, C) showed significant differences in metabolites between the tumor group and the control group. Besides, 200 permutations were conducted to validate the fitting degree of this model. Two components were selected for positive-ion metabonomic signatures (Fig. 4B, D), and the R2 value and Q2 value were 0.83 and -0.51 respectively. Two components were selected for negative-ion metabonomic signatures, and the R2 value and Q2 value were 0.91 and − 0.33, respectively. According to the results, the model was stable without over-fitting. The PLS-DA corroborated significant metabolomics differences between the two groups.

**Fig. 5:**
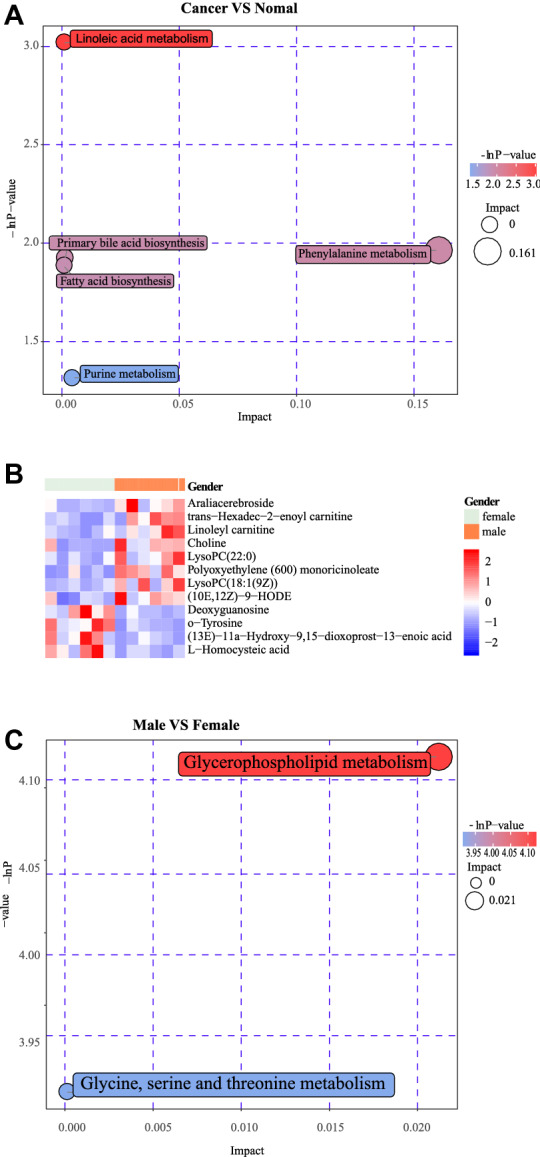
**A** The differently expressed metabolic pathway between colorectal cancer group and healthy people. **B** Heatmap showed the differentially expressed substances between colorectal cancer patients of different sex. **C** The bubble plot exhibited the pathway enriched by the differentially expressed substances between colorectal cancer patients of different sex

As shown in Fig. 5E, a lot of metabolites increased in the cancer group including 3-Methyladenine, N-Hydroxy-1-aminonaphthalene, Demethylated antipyrine, and so on. Amitenone Linoleamide, 5-Hydroperoxyeicosatetraenoic acid, Linoleyl carnitine, Deoxycholic acid 3-glucuronide decreased in the cancer group. In addition, Amitenone correlated with the expression of a variety of materials such as Linoleamide, 5-Hydroperoxyeicosatetraenoic acid, Linoleyl carnitine, and Deoxycholic acid 3-glucuronide (Fig. 4F). The differentially expressed metabolites were mainly enriched in the following KEGG pathways: Linoleic acid metabolism, Phenylalanine metabolism, Primary bile acid biosynthesis, Fatty acid biosynthesis, and Purine metabolism (Fig. 5A).

**Fig. 6:**
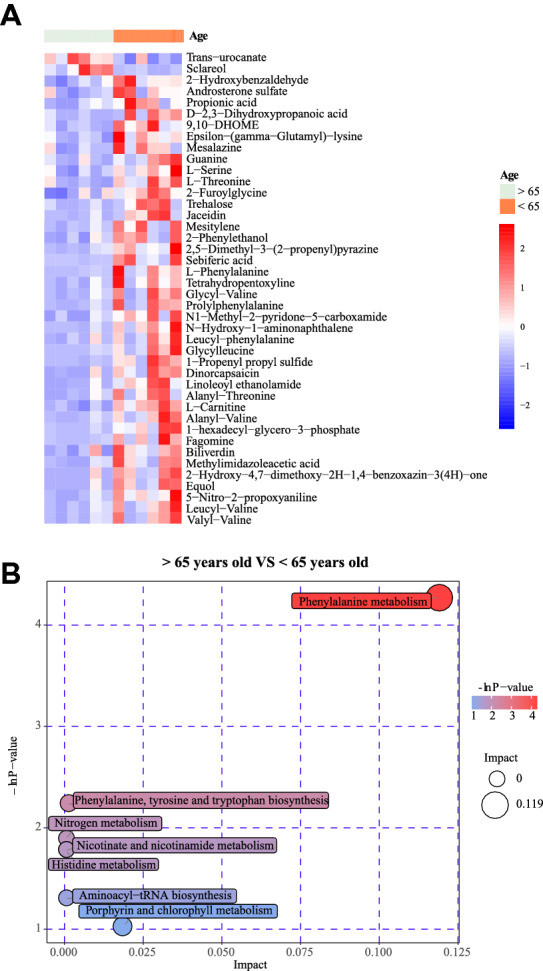
**A** Heatmap showed the differentially expressed substances between older patients and younger patients, and we set the cut-off point at 65. **B** The differently expressed metabolic pathway between colorectal cancer patients divided by 65 years old

Metabolomics studies of colorectal cancer patients with different genders and different ages were conducted, and the materials such as Choline polyoxaleate (600) monoricinoleate LysoPC(22:0) LysoPC(18:1(9Z)) Araliacerebroside trans-hexadec-2-enoyl carnitine Linoleyl carnitine, and other metabolites decreased in male patients with colorectal cancer (Fig. 5B). These substances are mainly enriched in the metabolic pathways including Glycerophospholipid metabolism Choline metabolism in cancer Glycine, and serine and threonine metabolism (Fig. 5C). Daidzein, Trigonelline, PC-M6, 5-hexyltetrahydro-2-furanoctanoic acid, m-aminobenzoic acid, Pyrophaeophorbide A, and all-trans-retinoic were more abundant in patients with colorectal cancer over 65 years old than colorectal cancer patients under 65 years old. (Fig. 6A). The differentially expressed metabolites were mainly enriched in the following KEGG pathways: Phenylalanine metabolism, Phenylalanine, tyrosine, and tryptophan biosynthesis, Nicotinate, and nicotinamide metabolism, Histidine metabolism, Aminoacyl-tRNA biosynthesis, Porphyrin and chlorophyII metabolism (Fig. 6B).

## Discussion

In this study, we found that the gut microbiota data of patients with colorectal cancer and the healthy control group was significantly different, for instance, the abundance of Blautia, Ruminococcus in colorectal cancer patients was significantly increased. In addition, we found that the abundance of Blautia Genus was positively correlated with the age of patients, and the abundance of Ruminococcus Genus of male patients was higher than that of female patients. However, our study did not discover obvious differences as for the amount of Lactobacillus genus, Ruminococcus genus, and Blautia Genus of colorectal cancer patients with different BMI.

To explore the possible mechanism of the impact of gut microbiota on the COVID-19, we collected 12 patients with colorectal cancer and 8 health control specimens, and we found a significant difference in metabolomics between patients with colorectal cancer and the health control specimen: 3-Methyladenine, N-hydroxy- 1-aminonaphthalene Demethylated antipyrine and other metabolites were differentially expressed. And there is some correlation between the expression of these metabolites. In addition, we also found that the alteration of these metabolites may be related to the alteration of some metabolic pathways.

Gut microbiota comprises a wide range of microorganisms that interact with host cells to regulate many physiological processes, such as energy harvest, metabolism, and immune response [26]. Gut microbiota may also contribute to a variety of respiratory infectious diseases, and this interaction effect was known as the gut-lung axis [5, 42, 43]. Gou et al. [10] found that the Blautia genus, Lactobacillus genus, and Ruminococcus genus of Firmicutes in the gut microbiota of COVID-19 patients correlated with the expression of multiple inflammatory molecules and that the operational taxonomic unit (OTU) composed of these three genera is associated with the severity of COVID-19. Therefore, it is of great significance to study the differences between these bacteria in health control people and patients with colorectal cancer as a risk factor of COVID-19. In this study, it was found that Blautia and Ruminococcus had a higher abundance in colorectal cancer patients, indicating that colorectal cancer patients have a higher tendency to severe COVID-19 infection. We found that Blautia genus had a higher concentration in older patients and females, which indicated that these two populations infected with COVID-19 have a higher likelihood of severe illness.

Previous studies have found that “cytokine storm”, excessive production of inflammatory cytokines, may be an important mechanism leading to an increase in the severity and mortality of COVID-19 patients [12]. In our work, IL-1β and IL-6 were increased in the intestinal tumors of mice after being fed by feces from colorectal cancer patients compared to counterparts fed by feces from healthy people. Previous studies also showed that Blautia and Ruminococcus were positively correlated with the expression of these two cytokines [8], and the pro-inflammatory status caused by the increase of IL-1β may cause the deterioration of patients with COVID-19.

Our study found significant differences in metabolomic results between patients with colorectal cancer and healthy controls. In the samples of colorectal cancer patients, Dodecanoic acid, 3-methyladenine, and other substances increased, while Inosine and other substances decreased. These substances were enriched in Linoleic acid (LA) metabolism, Phenylalanine metabolism, Fatty acid biosynthesis, Purine metabolism, and other pathways. Amitenone correlated with the differential expression of a variety of metabolites, and it might be important in the metabolism of gut microbiota, but its role in COVID-19 has not been reported before.

Using 2.85-angstrom cryo-electron microscopy, Christine et al. found that the receptor-binding domains tightly bind the essential free fatty acid LA in three composite binding pockets [44]. LA binding stabilizes a locked COVID-19 spike conformation, resulting in reduced ACE2 interaction in vitro. In human cells, LA supplementation synergizes with the COVID-19 drug remdesivir, suppressing SARS-CoV-2 replication. The addition of phenylalanine might affect COVID-19 mRNA cap-1 methyltransferase function [45]. The low purine diet (especially in patients with hyperuricemia) as adjuvant nutritional therapy improves the immune system, weakens viral replication, and assists in the treatment of COVID-19. Therefore, changes in Linoleic acid metabolism, Phenylalanine metabolism, and Purine metabolism pathways may lead to changes in the infection degree of COVID-19.

We also found that the metabolomic differences between male and female colorectal cancer patients were mainly concentrated in the metabolic pathways such as Glycerophospholipid metabolism, Glycine, serine, and threonine metabolism. Juanjuan Xu et al [46] found that the pulmonary function of COVID-19 survivors is related to the Glycophospholipid metabolic pathway. Gut microbiota may affect the prognosis of COVID-19 patients by affecting metabolic pathways such as the Glycophospholipid metabolic pathway. The metabolome differences between patients older than 65 years old and those younger than 65 years old are mainly reflected in Linoleic acid metabolism, Phenylalanine metabolism, and other pathways, which may also be the mechanism of gut microbiota playing a role in metabolism.

Although there are meaningful results of this research, there are also some limitations. First, We hypothesize that differences in gut microbiota between CRC and non-tumor groups may also lead to differences in mortality between them based on the previous studies, and we really discovered the differences in Blautia genus and Ruminococcus genus between CRC and non-tumor groups, but our hypothesis still needs further research to confirm. Our studies may provide directions for follow-up studies. Secondly, we analyzed abundant public data from GMrepo database, but the sample size difference between the healthy and patients with colorectal neoplasms in this dataset was too big. Although we used other datasets to validate our results, further studies are needed to confirm these discoveries.

This study indicated that the difference in the gut microbiota of colorectal cancer patients and healthy control people may lead to a worse prognosis in colorectal cancer patients with COVID-19. This study further illustrated the effect of gut microbiota in the severity and treatment of colorectal cancer and COVID-19, which might provide a new idea for the treatment of colorectal cancer patients with COVID-19. However, further studies are needed to validate the results of this study and to further explore the mechanism of the role of gut microbiota in COVID-19.

## Conclusion

Previoius studies showed that Blautia genus, Lactobacillus genus, and Ruminococcus genus of Firmicutes are correlated with the severity of COVID-19^10^. Based on the GMrepo database, We found that Blautia genus and Ruminococcus genus enrichment degress may be different between CRC patients and healthy controls, which may lead to a worse prognosis in colorectal cancer patients with COVID-19. In addition, We collected feces samples from 12 patients with colorectal cancer and 8 healthy people in Xiangya hospital for metabolomic analysis to investigate the potential mechanisms. Further studies are needed to validate the results of this study and explore the mechanism of the role of gut microbiota in COVID-19.

## Supplementary Information

**Additional file 1**:** Figure S1**. The expression of cytokines in the intestinal tumor of mice after beinggavaged by feces from colorectal cancer patients compared to healthy people based onGSE 136682 data, and IL-1β and IL-6 were increased.** Table S1**. The clinical features of the GMrepo cohort.** Table S2**. The clinical features of the validation cohort.

## Data Availability

The datasets used and analyzed during the current study are available from the corresponding author on reasonable request.

## Abbreviations

OTU: Operational taxonomic unit
ACE2: Angiotensin converting enzyme 2
GEO: Gene-Expression Omnibus
EMR: Electronic medical records
OPLS-DA: Supervised orthogonal projections to latent structures-discriminate analysis
VIP: Variable importance in the projection
CRC: Colorectal cancer
