# Supplementary material for: Gut microbiota imbalance in colorectal cancer patients, the risk factor of COVID-19 mortality
Source: Gut Pathog. 2021 Dec 4;13:70. doi: 10.1186/s13099-021-00466-w (PMC8643189; doi:10.1186/s13099-021-00466-w)
Supplement: Supplementary file 1 — Additional file 1: Figure S1. The expression of cytokines in the intestinal tumor of mice after beinggavaged by feces from colorectal cancer patients compared to healthy people based onGSE 136682 data, and IL-1β and IL-6 were increased. Table S1. The clinical features of the GMrepo cohort. Table S2. The clinical features of the validation cohort. [file 13099_2021_466_MOESM1_ESM.doc]

Figure S1


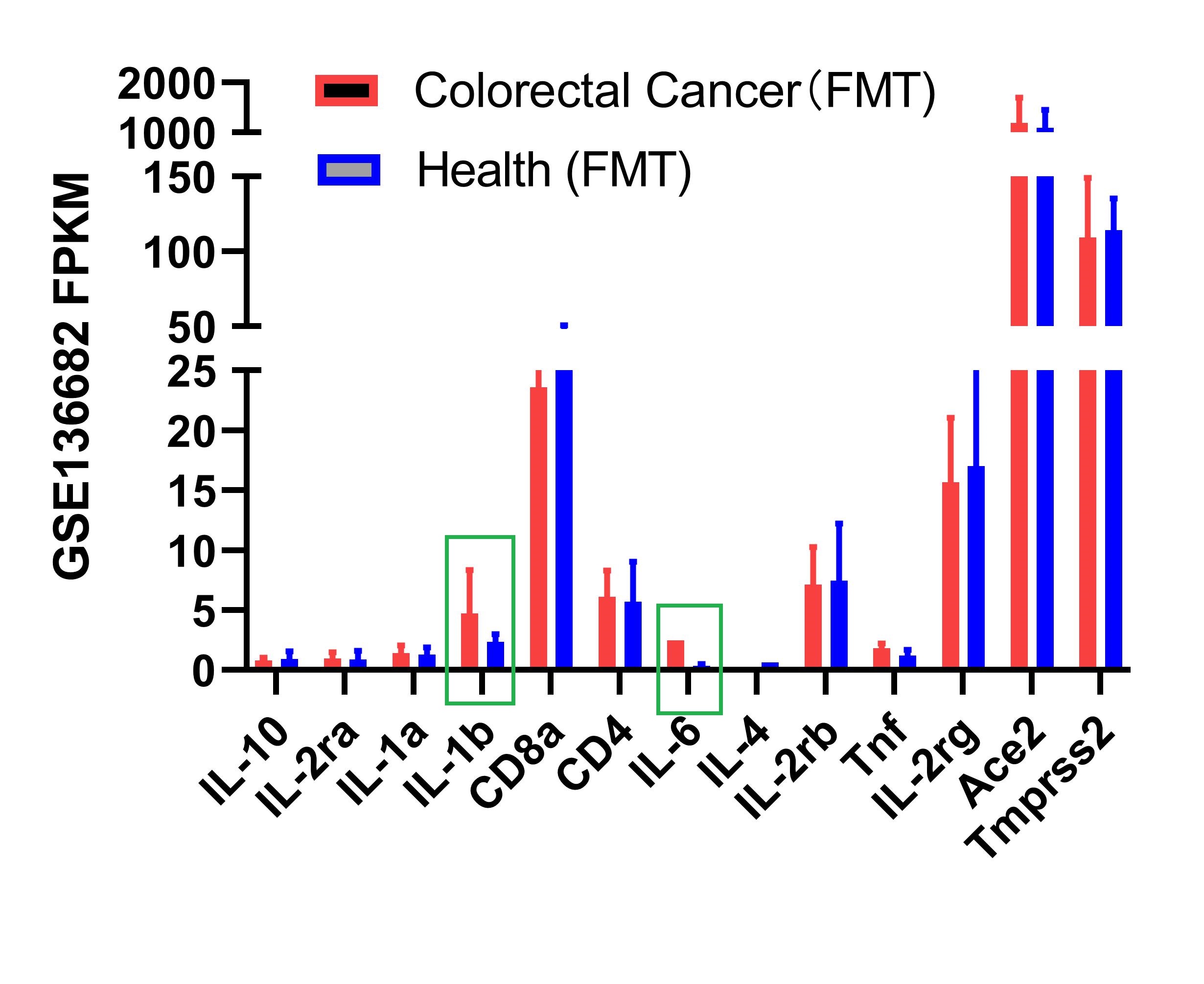


| Table S1. The clinical features of GMrepo cohort | | | | |
| --- | --- | --- | --- | --- |
| Group | Sub-group | Health (27329) | Colorectal Neoplasms (1374) | |
| Gender | Male | 7880 | 452 | |
|  | Female | 7754 | 315 | |
|  | unknown | 11695 | 607 | |
| Age | ≥65 | 1019 | | 423 |
|  | <65 | 10996 | 360 | |
|  | unknown | 15314 | 591 | |
| BMI | ≥31 | 256 | 93 | |
|  | ＜31 | 6474 | 578 | |
|  | unknown | 20599 | 703 | |
| Gut microbiota genus | Blautia | 11630 | 1233 | |
|  | Lactobacillus | 7763 | 614 | |
|  | Ruminococcus | 10619 | 1126 | |

Table S2. The clinical features of validation cohort

| Group | sub-group | colorectal cancer | Health | P value |
| --- | --- | --- | --- | --- |
| Gender | Male | 6 | 4 | 1 |
|  | Female | 6 | 4 | 1 |
|  |  |  |  |  |
| Age | ≥65 | 6 | 4 | 1 |
|  | ＜65 | 6 | 4 | 1 |
